# Ebola virus disease and maternal–neonatal health: direct and indirect impacts and health system preparedness gaps

**DOI:** 10.3389/fpubh.2026.1952690

**Published:** 2026-10-12

**Authors:** Nasro Salad Ahmed, Fatima Abdijabbar Ibrahim, Omar Aweis Ali

**Affiliations:** 1 Faculty of Health Sciences and Tropical Medicine, Somali National University, Mogadishu, Somalia; 2 Climate and Environmental Research Institute (CERI), Mogadishu, Somalia; 3 Center for Natural Resources, Universidade Estadual do Mato Grosso do Sul (UEMS), Dourados, Brazil

**Keywords:** Ebola virus disease, Ebola virus infection, fetal outcomes, health system disruption, infection prevention and control, maternal health services, maternal mortality, neonatal outcomes

## Abstract

**Background:**

Ebola virus disease (EVD) is a severe viral infection that can substantially affect maternal and neonatal health through both direct disease and disruption of essential health services. Pregnant women and neonates may experience serious adverse outcomes during outbreaks, yet pregnancy-specific evidence remains limited and maternal–neonatal health has historically received insufficient attention in Ebola preparedness and research.

**Methods:**

This narrative review synthesized evidence on Ebola virus disease and maternal–neonatal health from peer-reviewed literature, international guidance, and outbreak reports. Relevant literature was identified through targeted searches of PubMed, Scopus, and Google Scholar and through reference-list screening of key publications. Findings were synthesized narratively, with attention to study design, methodological limitations, and differences across outbreak settings.

**Findings:**

Available evidence indicates that EVD during pregnancy is associated with severe maternal complications and poor fetal and neonatal outcomes, although risk estimates remain uncertain because of small and heterogeneous studies. Placental infection and mother-to-child transmission have been documented. Ebola outbreaks also disrupt antenatal, delivery, immunization, and emergency obstetric services, contributing to additional maternal and neonatal morbidity and mortality. Key preparedness gaps include limited maternity-specific IPC, inadequate pregnancy-disaggregated surveillance, and insufficient evidence on vaccines and therapeutics in pregnancy.

**Conclusion:**

EVD poses serious but context-dependent risks to maternal and neonatal health through direct disease and disruption of essential healthcare services. Preparedness should prioritize continuity of essential maternity and newborn care, maternity-specific IPC, pregnancy-disaggregated surveillance, and the responsible inclusion of pregnant and breastfeeding women in vaccine and therapeutic research and access.

## 1 Introduction

One of the deadliest infectious diseases affecting human populations, Ebola virus disease (EVD) is caused by viruses that belong to the Ebolavirus genus. Since its first outbreaks were identified in 1976, EVD has caused recurrent epidemics, mostly in sub-Saharan Africa, with case fatality rates ranging from roughly 25 to 90% depending on viral species, outbreak characteristics, and healthcare capacity (1–3). In addition to the high mortality among infected individuals, Ebola outbreaks often overburden vulnerabilities in global health preparedness (1, 3, 4).

Pregnant women and newborns are a particularly vulnerable population during Ebola outbreaks because of the complex physiological and immunological adaptations that may influence host responses to severe viral infections, and maternity care environments involve frequent exposure to blood and body fluids, creating unique infection prevention challenges for healthcare workers (5, 6). Despite this vulnerability, pregnant women have historically been underrepresented.

The 2014–2016 West African Ebola epidemic marked a watershed moment in understanding the effects of EVD on maternal and newborn populations. “Although symptomatic maternal EVD was associated with serious adverse outcomes, the epidemic also demonstrated the substantial indirect effects of health-system disruption.” Fear of Ebola transmission, healthcare worker infections, and resource reallocation all contributed to lower rates of prenatal care attendance, skilled birth attendance, emergency obstetric services, and childhood vaccine coverage (7–9). These indirect repercussions demonstrated that Ebola’s impact goes beyond viral transmission to encompass broader shortcomings in maintaining key health services during emergencies.

Previous reviews have addressed important but relatively distinct aspects of EVD in pregnancy. Jamieson et al. focused primarily on the clinical and obstetric management of pregnant women with EVD and the occupational risks associated with maternity care, whereas Kourtis et al. emphasized pregnancy-specific clinical considerations and infection prevention and control. Bebell et al. subsequently synthesized published evidence on maternal and perinatal outcomes, including pregnancy loss, neonatal outcomes, and placental involvement. However, these reviews were largely based on small case series, historical outbreak reports, and limited pregnancy-specific data, and they focused predominantly on the direct clinical consequences of maternal EVD. The indirect effects of Ebola outbreaks on maternal and newborn health services, together with preparedness issues such as maternity-specific IPC, pregnancy-disaggregated surveillance, vaccination, therapeutics, and continuity of essential obstetric and neonatal care, have generally been considered separately. The present review addresses this gap by integrating direct maternal, fetal, and neonatal consequences with indirect health-system effects and preparedness deficiencies within a single maternal–neonatal health framework. This integrated approach provides a broader basis for identifying priorities for protecting pregnant women and newborns during future Ebola outbreaks.

As a result, the purpose of this narrative review is to critically synthesize current evidence on the direct and indirect effects of EVD on maternal and neonatal health, identify significant gaps in outbreak preparedness and pregnancy-specific evidence, and highlight priorities for improving maternal and neonatal health protection during future Ebola outbreaks (Figure 1).

**Figure 1:**
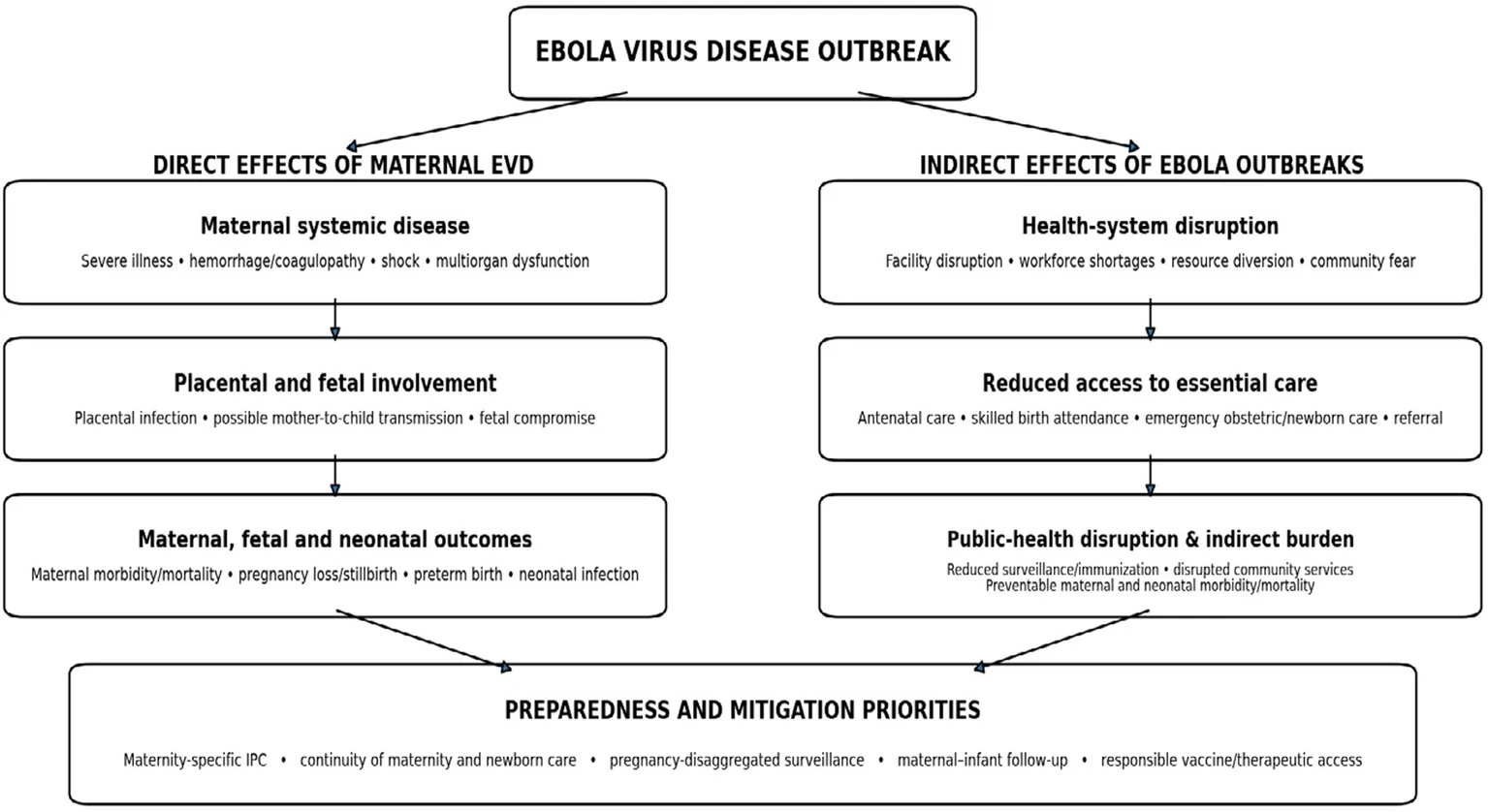
Conceptual framework illustrating the direct and indirect pathways through which Ebola virus disease may affect maternal and neonatal health.

## 2 Methodology

### 2.1 Overview

This study employed a narrative review methodology guided by the Scale for the Assessment of Narrative Review Articles (SANRA) to enhance transparency, methodological rigor, and critical synthesis. The review examined the direct and indirect effects of Ebola virus disease (EVD) on maternal and neonatal health, including clinical outcomes, health-service disruption, and preparedness gaps. A narrative approach was appropriate because the available evidence includes heterogeneous observational studies, outbreak reports, case series, modeling studies, reviews, and public-health guidance.

#### 2.1.1 Literature identification and selection

Relevant literature was identified through targeted searches of PubMed, Scopus, and Google Scholar, together with examination of the reference lists of key reviews and primary studies. Searches focused on combinations of terms related to Ebola virus disease, pregnancy, maternal health, fetal and neonatal outcomes, vertical transmission, infection prevention and control, health-service disruption, vaccination, therapeutics, surveillance, and preparedness.

The purpose of the search was to identify literature relevant to the objectives of this narrative review rather than to conduct an exhaustive or systematic search of all available evidence. Publications were selected on the basis of their relevance to maternal, fetal, neonatal, placental, and health-system aspects of Ebola virus disease. Because this was a narrative review, a formal systematic-review protocol, PRISMA-based screening process, and database-specific reproducible search strategy were not applied.

#### 2.1.2 Inclusion and exclusion criteria

Publications were included if they reported maternal, fetal, neonatal, placental, or pregnancy-related outcomes associated with EVD; examined disruption of maternal and child health services; or addressed outbreak preparedness, IPC, surveillance, vaccination, or therapeutics. Relevant observational studies, outbreak investigations, case reports/series, modeling studies, reviews, and authoritative public-health guidance were considered.

Publications were excluded if they were duplicates, outside the specified period, unavailable in English, unrelated to maternal–neonatal health, or lacked sufficient substantive information for interpretation.

#### 2.1.3 Data extraction and synthesis

Relevant information was extracted according to major themes: EVD epidemiology in pregnancy, maternal vulnerability, placental and vertical transmission, maternal outcomes, fetal and neonatal outcomes, disruption of maternal and child health services, and health-system preparedness.

Because of substantial heterogeneity in study designs, populations, outbreak settings, sample sizes, and reported outcomes, meta-analysis was not considered appropriate. Findings were therefore synthesized narratively through thematic comparison, with emphasis on consistent patterns, conflicting evidence, contextual differences, and remaining knowledge gaps.

#### 2.1.4 Quality assessment of sources

The overall quality and reporting of the review were guided by SANRA principles. Included evidence was critically evaluated according to study design, outbreak setting, sample size, population definition, case definition, outcome ascertainment, availability of comparison groups, and whether estimates were directly observed or model-derived.

No single formal risk-of-bias tool was applied because of the substantial heterogeneity of the evidence base. Greater interpretive weight was given to studies with clearly defined populations, transparent denominators, appropriate comparison groups, and well-documented outcomes, while small case series, historical reports, and model-based estimates were interpreted cautiously.

## 3 Epidemiology of Ebola virus disease and pregnancy

Ebola virus disease (EVD) is a serious zoonotic illness characterized by periodic outbreaks, high case fatality rates, and significant disruption of healthcare systems, particularly in areas with low capacity. Since the first known outbreaks in 1976 in the Democratic Republic of the Congo (DRC) and Sudan, EVD outbreaks have varied greatly in terms of magnitude, epidemiological characteristics, viral species, and health-care setting (1, 10).

Between 2014 and 2016, West Africa saw the greatest documented EVD outbreak, which mostly affected Guinea, Liberia, and Sierra Leone. More than 28,000 cases and 11,000 deaths were documented, posing an extraordinary burden to afflicted healthcare systems (4). In addition to EVD-related morbidity and mortality, the epidemic impacted routine healthcare delivery, including reproductive, maternal, neonatal, and child health services (7–9).

Pregnancy-specific epidemiological evidence is sparse in comparison to the overall EVD burden. Despite this, historical records show that women with symptomatic EVD have poor maternal and pregnancy outcomes. During the 1995 Kikwit outbreak in the DRC, Mupapa et al. reported EVD in 15 pregnant women, 14 of whom died, for a maternal case fatality rate of roughly 93% (11). Pregnancy loss was also common among this tiny historical group. However, these findings should be interpreted with caution due to the small sample size and the clinical and outbreak settings at the time.

Evidence gathered during the 2014–2016 West African epidemic revealed more significant outcomes among pregnant women tested for EVD, while estimates varied depending on the research population and outcome definition (12, 13). Importantly, most accessible research focused on women with symptomatic or suspected EVD rather than population-based samples of all pregnant women infected with Ebola virus. As a result, stated maternal, fetal, and neonatal outcome estimates should not be applied to all illnesses during pregnancy.

The epidemiology of pregnancy-related EVD is also influenced by outbreak-specific and health-system factors. Delayed diagnosis, restricted access to specialized obstetric and supportive care, difficulties adopting maternity-specific infection prevention and control, and interruption of referral routes all have the potential to influence clinical outcomes. Fear of healthcare-associated infection, health-worker shortages, facility closures, and resource diversion can all restrict access to prenatal, delivery, and other critical maternal and child health services (8, 9, 14). These indirect pathways must be separated from those affected directly by maternal EVD.

Significant variation between outbreaks further limits the evaluation of pregnancy-specific risk. Variations in reported outcomes may be due to differences in viral species, outbreak volume and duration, baseline health-system capacity, surveillance, clinical management, and vaccine and treatment availability. As a result, historical estimates and findings from the 2014–2016 West African outbreak should be evaluated within their specific epidemiological and healthcare contexts, rather than as general estimates of pregnancy-associated EVD risk.

Although the overall number of confirmed pregnancy-associated EVD cases is still minimal, the severity of reported outcomes and the complexities of obstetric therapy make maternal and neonatal health a crucial component of epidemic response (Table 1).

**Table 1:** Characteristics and key findings of major studies on Ebola virus disease and maternal–neonatal health.

Study	Country/outbreak	Study design	Population/sample size	Main findings/quantitative estimate	Evidence limitations
Mupapa et al. (1999) (11)	Democratic Republic of the Congo; Kikwit outbreak, 1995	Observational case series	15 pregnant women with EVD	14 of 15 women died, corresponding to a maternal case-fatality proportion of approximately 93%. Pregnancy loss was also frequently reported.	Very small historical cohort; clinical management and supportive care differed substantially from contemporary EVD care; limited generalizability.
Jamieson et al. (2014) (5)	Evidence available during the 2014 West African outbreak	Clinical review	Pregnancy-associated EVD evidence available at the time	Highlighted severe maternal and pregnancy outcomes, obstetric-management challenges, and substantial occupational exposure risks during maternity care.	Predominantly based on historical reports, case series, and limited pregnancy-specific evidence; no pooled quantitative risk estimate.
Kourtis et al. (2015) (6)	Historical and West African outbreak evidence	Clinical review	Published pregnancy-related EVD evidence	Identified pregnancy-specific clinical and IPC challenges and emphasized the limited evidence available to guide obstetric management.	Evidence was largely derived from small case reports, case series, and outbreak observations.
Bebell et al. (2017) (12)	Multiple historical and West African outbreaks	Narrative review	111 published pregnancy-associated EVD cases	Reported an overall maternal mortality proportion of approximately 86% across published pregnancy-associated cases and documented substantial fetal and neonatal morbidity and mortality.	Cases were drawn from heterogeneous outbreaks and clinical settings; substantial selection and reporting bias; historical estimates should not be interpreted as universal risks.
Lyman et al. (2018) (13)	Sierra Leone; 2014 outbreak	Observational investigation	192 pregnant women with suspected EVD; 67 EVD-positive	Among women with known maternal outcomes, 53/65 EVD-positive women died compared with 35/116 EVD-negative women; OR 10.22 (95% CI 4.87–21.46). Spontaneous abortion occurred in 14/67 EVD-positive pregnancies versus 6/118 EVD-negative pregnancies; OR 4.93 (95% CI 1.79–13.55). Among documented delivery outcomes, 9/15 births among EVD-positive women were stillbirths, and 5/6 live-born neonates subsequently died.	Clinically selected population of women with suspected EVD; several fetal and neonatal outcomes involved small denominators; findings should not be generalized to all pregnant women infected with Ebola virus.
Sochas et al. (2017) (7)	Sierra Leone; 2014–2016 Ebola epidemic	Modeling study based on reductions in reproductive, maternal, and infant health-service utilization	Population-level model	Estimated approximately 3,593–4,936 additional maternal deaths, neonatal deaths, and stillbirths associated with reduced utilization of essential maternal and infant health services during the epidemic.	Estimates were model-derived rather than directly observed and depended on assumptions regarding service utilization and the Sierra Leone health-system context.
Delamou et al. (2017) (8)	Guinea; 2014–2016 Ebola epidemic	Retrospective observational cohort study	Maternal and child health-service data from Ebola-affected health districts	Demonstrated substantial reductions in antenatal-care attendance, institutional delivery, and other maternal and child health services during the Ebola epidemic.	Health-service effects were context-specific; observational design limits causal attribution of all changes directly to Ebola.
Ribacke et al. (2016) (9)	West African Ebola epidemic	Systematic review	Studies examining healthcare utilization during the epidemic	Reported consistent reductions in healthcare utilization, including maternal health services, facility-based care, and routine child-health services during the outbreak.	Considerable heterogeneity across countries, health services, study designs, and outbreak periods; magnitude of disruption varied by

## 4 Pathophysiology and pregnancy-specific vulnerability

Ebola virus illness is defined by the interconnections of viral replication, immunological dysregulation, endothelial dysfunction, and coagulation abnormalities. Following infection, the Ebola virus targets a variety of cell types, including monocytes, macrophages, and dendritic cells, causing compromised innate and adaptive immune responses, systemic inflammation, vascular dysfunction, coagulation abnormalities, and progressive multiorgan involvement (1, 15).

Pregnancy causes dynamic immunological and physiological changes that can affect the clinical course of serious infectious illnesses. Changes in maternal immune modulation, together with cardiovascular, hematological, and metabolic changes, may influence the maternal response to EVD (5, 6). However, the extent to which pregnancy-specific physiological changes independently affect the severity of EVD is unclear, and available findings should be read with caution.

Placental involvement is a key aspect of EVD during pregnancy. In recorded cases, Ebola virus or viral material was found in placental and fetal-associated tissues, indicating that placental infection and mother-to-child transmission are possible (5, 6, 11, 12). Placental involvement may contribute to inflammatory damage and disruption of normal placental function, although the precise mechanisms linking placental infection to specific fetal outcomes remain poorly understood.

To clarify, maternal EVD, placental infection, fetal infection, and vertical transmission are not interchangeable. Maternal EVD refers to clinically apparent disease in pregnant women, whereas placental infection denotes viral involvement of placental tissues. Vertical transmission occurs from the mother to the fetus or neonate, and neonatal infection can result from intrauterine, intrapartum, or postnatal exposure. Available case reports and outbreak observations suggest that placental and fetal involvement occurs in certain pregnancies affected by symptomatic maternal EVD; nonetheless, the frequency, timing, and factors of mother-to-child transmission remain poorly characterized (12, 13). These ambiguities limit the ability to draw solid conclusions on the mechanisms driving fetal loss, stillbirth, and neonatal infection.

## 5 Direct maternal outcomes of Ebola virus disease during pregnancy

Symptomatic Ebola virus disease (EVD) during pregnancy has been associated with serious maternal morbidity and mortality, although the magnitude of risk has varied across outbreaks and study populations. Much of the available evidence derives from small historical cohorts, case series, and observational investigations, which limits the precision and generalizability of pregnancy-specific risk estimates (5, 6, 11–13).

Early findings from the 1995 Kikwit outbreak in the Democratic Republic of the Congo revealed very bad maternal outcomes. 14 of 15 pregnant women with EVD died, resulting in a maternal case fatality rate of roughly 93% (11). A later synthesis of 111 published pregnancy-associated EVD cases found an overall maternal mortality rate of roughly 86%, despite the fact that the cases included were from various outbreaks, time periods, and clinical settings (12). These historical findings should not be regarded as general estimates of maternal mortality among pregnant women with EVD.

Evidence from the 2014 West African outbreak revealed more catastrophic maternal outcomes, but also showed diversity between contexts. In a Sierra Leone observational study of 192 pregnant women with suspected EVD, 67 tested positive for EVD. Among women with known maternal outcomes, 53 of 65 EVD-positive women died (81.5%), versus 35 of 116 EVD-negative women (30.2%). EVD positive was associated with a significantly higher risk of maternal death (OR 10.22, 95% CI 4.87–21.46) (13). Because this study included pregnant women with probable EVD, these estimates largely describe a clinically selected cohort and should not be applied to all pregnant women with Ebola virus.

Severe maternal illness can cause systemic inflammation, coagulation problems, shock, hemorrhagic complications, and multiorgan failure (1, 15). Pregnancy-related physiological modifications may complicate clinical assessment and management; nevertheless, it is unclear how much pregnancy enhances EVD severity on its own. Obstetric problems, such as spontaneous abortion, fetal death, premature labor, and bleeding caused by pregnancy loss or delivery, might complicate care (5, 6, 11–13, 19).

Clinical management poses significant infection prevention and control issues. Labor, delivery, and pregnancy loss management may include considerable contact with blood, amniotic fluid, placental tissue, and other body fluids, necessitating the use of maternity-specific IPC procedures and personal protective equipment to prevent occupational exposure (4–6).

Although supportive care and EVD therapies have evolved significantly since previous outbreaks, pregnancy-specific evidence is still more restricted than evidence for the general population (17, 18). Historically, pregnant women have been excluded or underrepresented in clinical research, leading to confusion about pregnancy-specific safety, efficacy, effectiveness, and appropriate clinical treatment. As a result, current results should not be considered to be identical to those recorded in previous outbreaks, especially where enhanced supportive care and effective medicines are available.

## 6 Direct fetal and neonatal outcomes associated with maternal EVD

Symptomatic maternal EVD has been associated with serious adverse pregnancy and neonatal outcomes, including spontaneous abortion, fetal death, stillbirth, preterm delivery, and neonatal infection (5, 6, 11–13). However, the available evidence is based largely on relatively small numbers of documented pregnancies, and the magnitude of these risks varies across outbreaks and clinical settings.

Quantitative evidence from Sierra Leone illustrates the increased risk of adverse pregnancy outcomes among women evaluated for suspected EVD. In an observational investigation of 192 pregnant women, spontaneous abortion occurred in 14 of 67 EVD-positive pregnancies (20.9%) compared with 6 of 118 EVD-negative pregnancies (5.1%), corresponding to an OR of 4.93 (95% CI 1.79–13.55) (13). Among the smaller subset with documented delivery outcomes, 9 of 15 births among EVD-positive women were stillbirths. Five of six live-born neonates following EVD-positive pregnancies subsequently died (13). These small denominators warrant considerable caution and preclude interpretation of these proportions as universal estimates of fetal or neonatal mortality.

Several mechanisms may contribute to adverse fetal and neonatal outcomes. Ebola virus or viral material has been detected in placental and fetal-associated tissues, supporting placental involvement and the possibility of mother-to-child transmission (11, 12). Placental infection may contribute to inflammatory injury and impaired placental function, while severe maternal illness including fever, shock, coagulation abnormalities, and multiorgan dysfunction may independently compromise fetal well-being. However, the relative contribution of direct fetal infection, placental dysfunction, and severe maternal systemic disease to individual pregnancy outcomes remains incompletely characterized.

The timing of maternal disease may also influence pregnancy outcome, but available data are insufficient to establish precise gestational-age-specific risks. Pregnancy loss has been reported following EVD at different stages of gestation, while fetal infection, stillbirth, preterm delivery, and neonatal infection have also been documented (11–13). Accordingly, statements that particular gestational periods consistently lead to specific outcomes should be interpreted cautiously until larger pregnancy-specific datasets become available.

Neonatal infection has been documented following maternal EVD, but the route and timing of transmission cannot always be established. Infection may potentially occur in utero, during delivery, or through postnatal exposure to infectious maternal body fluids. Therefore, maternal EVD, placental infection, fetal infection, vertical transmission, and neonatal infection should not be treated as interchangeable outcomes. Although mortality among reported infected or exposed neonates has been substantial in historical case reports and small series, the limited number of documented cases prevents precise estimation of neonatal mortality risk.

Overall, the existing evidence suggests a high risk of bad fetal and neonatal outcomes in pregnancies complicated by symptomatic maternal EVD, although there is significant uncertainty about the extent and determinants of that risk. Improved pregnancy-specific surveillance, systematic maternal–infant follow-up, and the inclusion of pregnancy and neonatal outcomes in outbreak research are required to generate more reliable estimates and determine how modern supportive care, vaccination, and therapeutics may influence these outcomes (Table 2).

**Table 2:** Direct pathobiological effects and clinical outcomes associated with maternal Ebola virus disease.

Domain	Mechanism	Main clinical consequences	Evidence consideration
Maternal systemic disease	Viral replication, immune dysregulation, endothelial and coagulation abnormalities	Severe illness, hemorrhage, shock, and multiorgan dysfunction	Well established in EVD, but pregnancy-specific effects remain incompletely defined
Placental and fetal involvement	Placental infection and possible mother-to-child transmission	Pregnancy loss, fetal compromise, stillbirth, and neonatal infection	Transmission is documented, but its frequency, timing, and mechanisms remain uncertain
Maternal illness–fetal compromise	Fever, shock, coagulation abnormalities, and severe systemic disease	Preterm delivery, fetal distress, pregnancy loss, or fetal death	Direct viral effects are difficult to separate from consequences of severe maternal illness
Obstetric management and IPC	Exposure to infectious blood, amniotic fluid, and placental tissues during care	Increased occupational exposure risk	Requires appropriate PPE, triage, isolation, and maternity-specific IPC

## 7 Indirect maternal and child health effects of Ebola outbreaks

The impact of Ebola virus disease goes beyond direct infection-related mortality to include significant indirect repercussions such as disruption of important maternal and child health care. During significant epidemics, health systems in afflicted countries frequently face concurrent challenges from increasing demand for Ebola-related care, healthcare worker illnesses, community fear, and the diversion of limited resources to emergency response efforts (7–9).

One of the most notable implications is a decline in the use of routine maternal health treatments. Evidence from the 2014–2016 West African Ebola pandemic revealed significant decreases in prenatal care attendance, facility-based births, skilled birth attendance, and access to emergency obstetric care (7, 9). Fear of contracting Ebola in healthcare facilities, along with temporary closure or limited functionality of health institutions, prompted many pregnant women to forego official care and give birth at home without trained support.

These disruptions may raise the risk of preventable maternal and newborn problems such as obstructed labor, postpartum hemorrhage, gestational hypertension, neonatal difficulties, and delayed emergency referral. In areas where maternal mortality was already high, even temporary cuts to key services may contribute to greater maternal and newborn mortality.

Child health services were also impacted. Routine immunization efforts were disrupted during Ebola outbreaks, resulting in lower vaccination coverage and greater vulnerability to vaccine-preventable illnesses (8, 9). Furthermore, nutritional programs, neonatal care services, and community health activities were regularly hampered by personnel shortages and operational constraints.

Observational research show that mother and child health services are significantly disrupted, whereas modeling studies quantify the potential mortality consequences of these disruptions. Sochas et al. (7) projected an additional 3,593–4,936 maternal deaths, neonatal deaths, and stillbirths in Sierra Leone as a result of reduced reproductive, maternal, and infant healthcare utilization during the Ebola epidemic.

Taken together, our findings show that Ebola epidemics can have a significant impact on mother and child health by disrupting key healthcare services, in addition to the direct effects of EVD. Outbreak preparedness should include measures for ensuring the continuity of maternal, newborn, and child health care, as well as Ebola case detection, treatment, isolation, and infection prevention and control (Table 3).

**Table 3:** Direct and indirect effects of Ebola virus disease on maternal and neonatal health.

Area	Direct effects associated with maternal EVD	Indirect effects associated with Ebola outbreaks
Maternal health	Severe systemic illness, hemorrhagic complications, shock, coagulation abnormalities, and multiorgan dysfunction	Reduced antenatal care, facility-based delivery, skilled birth attendance, and emergency obstetric care
Fetal and neonatal health	Placental involvement, pregnancy loss, fetal infection, stillbirth, and neonatal infection	Poorer neonatal outcomes due to reduced access to skilled delivery, newborn care, and referral services
Healthcare services	Increased occupational exposure risk among maternity healthcare workers through contact with infectious blood and body fluids	Facility disruption, workforce shortages, interrupted referral pathways, and reduced availability of essential services
Public health systems	Need for rapid case identification, isolation, specialized clinical management, and maternity-specific IPC	Disruption of routine surveillance, immunization, community-based care, and other essential health programs

## 8 Infection prevention and control in maternity care during Ebola outbreaks

Maternity care environments present specific infection prevention and control (IPC) challenges during Ebola outbreaks because labor, delivery, and management of pregnancy complications can involve extensive contact with blood, amniotic fluid, placental tissue, and other potentially infectious body fluids. Obstetric healthcare workers, including midwives and nurses, may face substantial occupational exposure risks when appropriate protective measures are unavailable or inconsistently implemented (4–6).

Effective IPC requires adaptation of routine maternity practices to outbreak conditions. Key measures include early screening and triage of pregnant women, timely identification of suspected EVD cases, appropriate isolation procedures, consistent availability and correct use of personal protective equipment (PPE), and specialized IPC training for maternity healthcare workers (4, 5).

Implementation of these measures can be particularly challenging in resource-constrained settings. Reported barriers include inadequate PPE supplies, limited isolation capacity, shortages of trained healthcare personnel, and weak referral pathways. During previous Ebola outbreaks, healthcare worker infections and associated workforce shortages further constrained service delivery and contributed to community concerns regarding the safety of healthcare facilities.

Based on the evidence synthesized in this review, maternity-specific IPC should be considered within broader national outbreak preparedness frameworks. Potential strategies include dedicated obstetric assessment and isolation pathways, simulation-based training for maternity teams, reliable access to PPE, and integration of maternal and reproductive health expertise into outbreak response structures. Implementation should take into account workforce capacity, infrastructure, supply availability, referral systems, and local resource constraints.

## 9 Surveillance and health system preparedness gaps

Despite the recognized vulnerability of pregnant women during Ebola outbreaks, pregnancy-specific surveillance remains insufficient in many affected settings. Routine Ebola surveillance systems often prioritize case detection and transmission monitoring but fail to systematically capture pregnancy status, gestational age, maternal outcomes, and neonatal consequences.

The absence of standardized pregnancy-related indicators limits the ability of public health authorities to accurately estimate disease burden and rapidly allocate appropriate resources. Important outcomes, including miscarriage, stillbirth, maternal complications, and neonatal infection, may therefore remain underreported.

Integrating pregnancy variables into existing outbreak surveillance platforms, including national Integrated Disease Surveillance and Response (IDSR) systems, would allow more timely identification of maternal and neonatal risks. Such systems should include:

- pregnancy status;
- gestational age;
- pregnancy outcomes;
- delivery location;
- maternal and neonatal survival outcomes.

Improved surveillance would support targeted interventions, strengthen resource allocation, and ensure that maternal and neonatal populations are considered during early outbreak planning.

## 10 Vaccines and therapeutics: evidence gaps in pregnancy

The evidence supporting Ebola vaccines and therapeutics differs substantially between the general population and pregnant and breastfeeding women. Although vaccines and monoclonal antibody therapies have demonstrated efficacy or effectiveness in broader populations, pregnancy-specific evidence remains comparatively limited. General-population efficacy or effectiveness should therefore not be interpreted as direct evidence of equivalent pregnancy-specific efficacy, effectiveness, or safety. The development of Ebola vaccines and therapeutics has significantly improved outbreak control; however, pregnant women have historically benefited less from these advances because of delayed inclusion in clinical research.

The recombinant vesicular stomatitis virus-based Ebola vaccine (rVSV-ZEBOV) demonstrated high effectiveness during the Guinea ring vaccination trial and became a cornerstone of Ebola prevention strategies (16). However, pregnant and breastfeeding women were initially excluded from major clinical trials because of concerns regarding theoretical fetal risks associated with vaccine platforms. Following accumulation of safety data and recognition of the exceptionally high risk of Ebola infection during pregnancy, international recommendations evolved to support vaccination of pregnant and lactating women during Ebola outbreaks when benefits outweigh potential risks (2, 14). Similarly, monoclonal antibody therapies, including approved treatments such as Inmazeb and Ebanga, have improved survival among individuals with EVD (16). Nevertheless, pregnancy-specific pharmacokinetic data, safety monitoring, and clinical effectiveness evidence remain limited.

The historical exclusion of pregnant women from infectious disease research represents a major evidence gap. Future Ebola vaccine and therapeutic trials should incorporate ethical frameworks that enable responsible inclusion of pregnant populations rather than systematically excluding them from potentially life-saving interventions.

## 11 Discussion

This narrative review focuses on the complex link between EVD and maternal and neonatal health. According to available information, pregnancies complicated by symptomatic maternal EVD can result in catastrophic maternal, fetal, and neonatal outcomes, whereas Ebola epidemics can interrupt critical reproductive, maternal, newborn, and child health services at the same time.

The existing evidence suggests a link between symptomatic maternal EVD and substantial poor pregnancy outcomes, but the extent of these risks is unclear. Historical reports have reported significant maternal death, pregnancy loss, stillbirth, and newborn mortality; however, much of this information is based on tiny case series, selected symptomatic populations, and outbreak-specific studies. As a result, previous estimates should not be taken as universal risks that apply to all pregnant women with Ebola virus infection.

Placental involvement has been documented in pregnancies affected by maternal EVD and may contribute to adverse fetal and neonatal outcomes (5, 6, 11, 12). Nevertheless, maternal EVD, placental infection, fetal infection, vertical transmission, and neonatal infection represent distinct clinical and biological phenomena. The relative contributions of placental infection, severe maternal systemic illness, gestational timing, and other clinical factors to specific pregnancy outcomes remain incompletely characterized.

An equally noteworthy result is the significant indirect burden associated with health-care disruption. Observational studies conducted during the 2014–2016 West African epidemic revealed declines in antenatal care, facility-based delivery, skilled birth attendance, children immunization, and other critical services (7–9). Modeling studies have indicated higher maternal and neonatal mortality and stillbirth rates associated with decreased healthcare utilization. However, these mortality estimates are model-derived and context sensitive, and should not be taken as proving that indirect mortality always equals or exceeds mortality directly attributed to EVD.

The interpretation of pregnancy-associated EVD outcomes must take into account significant variation among outbreaks. Differences in viral species, outbreak magnitude and length, baseline health-system capability, surveillance quality, supportive and obstetric care availability, and vaccine and treatment access may all have an impact on reported maternal and neonatal outcomes. Findings from previous outbreaks and the 2014–2016 West African epidemic should not be applied to future outbreaks or different healthcare settings.

The study also shows that pregnant women and newborns face continuous readiness issues. Maternity services necessitate IPC procedures that allow for obstetric exposure to potentially infected bodily fluids while maintaining availability to emergency care. Pregnancy-disaggregated surveillance remains critical for producing accurate estimates of maternal and neonatal outcomes. At the same time, limited pregnancy-specific vaccine and therapeutic evidence continues to constrain evidence-based decision-making.

These findings support a preparedness approach that integrates maternal and neonatal health into outbreak response rather than treating maternity services as secondary to pathogen-control activities. The recommendations proposed in this review including strengthening pregnancy-specific surveillance, maintaining maternity-specific IPC and service continuity, and supporting responsible inclusion in vaccine and therapeutic research represent priorities derived from the overall narrative synthesis rather than direct empirical findings from any single study.

## 12 Limitations

This review has a few restrictions. First, as a narrative review, it lacked a formal systematic-review process, prospective PRISMA-style record accounting, and quantitative meta-analysis. Although the literature search was conducted across various databases and reinforced with relevant public-health guidelines, the lack of rigorous systematic screening may have resulted in selection and publication bias.

Second, the evidentiary basis for pregnancy-related EVD remains weak. Many available research include case reports, small case series, observational investigations, and outbreak-specific analyzes, which frequently have tiny denominators and inadequate follow-up. These factors reduce the precision of maternal, fetal, and neonatal risk estimations and the ability to draw causal inferences.

Third, there is significant variability between outbreaks in terms of virus species, clinical management, healthcare infrastructure, monitoring, supportive care availability, and access to vaccinations and therapies. A significant percentage of the current evidence stems from historical outbreaks and the 2014–2016 West African pandemic; thus, these findings may not be directly applicable to all contemporary or future outbreaks.

Fourth, the restriction on English-language publications may have created language bias and underestimated evidence from Francophone countries, particularly the Democratic Republic of the Congo and Guinea. This restriction should be taken into account when interpreting the geographical balance and generalizability of the evidence pooled in this review.

Finally, due to the narrative approach and the variety of the collected evidence, no formal risk-of-bias tool was used. Instead, evidence was assessed qualitatively based on study design, context, sample size and denominator, case definition, outcome assessment, the presence of comparison groups, and whether estimates were directly observed or model-derived. Nonetheless, this methodology does not provide the same standardized assessment of methodological quality as a systematic review.

## 13 Future research priorities

Future research should prioritize three areas that directly address the key gaps in evidence revealed in this assessment.

### 13.1 Pregnancy-specific surveillance and prospective registrations

During Ebola outbreaks, national health ministries, epidemic surveillance teams, maternity institutions, and foreign response partners should implement standardized systems for gathering pregnancy-specific data. The core variables should be pregnancy status, gestational age, illness severity, maternal outcome, pregnancy outcome, and newborn outcome. Prospective pregnancy registries and connected maternal–infant follow-up should enhance assessment of pregnancy-specific risks and allow for comparisons across epidemics. Important challenges to implementation include incomplete follow-up, limited surveillance personnel, data-quality limits, and issues integrating maternal and newborn records during emergencies.

### 13.2 Responsible inclusion in vaccine and therapeutic research

Research sponsors, regulatory authorities, ethics committees, ministries of health, and international organizations should create structures that allow pregnant and breastfeeding women to participate in vaccine and therapeutic research in a scientific and ethically sound manner. Pregnancy-specific pharmacovigilance, prospective safety monitoring, and maternal–infant follow-up should be included in intervention implementation where possible. Key problems include ethical and regulatory considerations, a limited number of eligible participants, difficulties sustaining follow-up during outbreaks, and uncertainty about prenatal and neonatal outcomes.

### 13.3 Maternity service continuity and IPC preparedness

Implementation research should evaluate feasible strategies for maintaining safe maternity and newborn services during Ebola outbreaks. Ministries of health, maternity facilities, WHO, and humanitarian partners should assess maternity-specific triage and isolation pathways, simulation-based IPC training, referral systems, PPE availability, and mechanisms for maintaining emergency obstetric and newborn care. Research should identify approaches that remain feasible in settings characterized by workforce shortages, infrastructure limitations, supply-chain disruption, and community mistrust.

## 14 Policy implications

Based on the evidence synthesized in this narrative review, three policy priorities warrant particular attention.

### 14.1 Incorporate pregnancy-specific surveillance into outbreak response

National health ministries and outbreak surveillance programs should systematically collect pregnancy status, gestational age, maternal outcome, pregnancy outcome, and newborn outcome as part of Ebola monitoring systems. Standardized data fields and connected maternal–infant follow-up would yield more solid evidence for clinical decision-making and resource allocation. To reduce new reporting obligations, implementation should leverage current surveillance platforms if practicable.

### 14.2 Protect continuity and safety of maternity and newborn services

Ministries of health, healthcare facilities, WHO, and humanitarian response partners should incorporate maternity-specific IPC and service-continuity plans into outbreak preparedness. Priority measures include safe obstetric triage, dedicated assessment and isolation pathways, reliable PPE availability, trained maternity teams, functioning referral systems, and mechanisms for maintaining emergency obstetric and newborn care. Resource constraints, workforce shortages, infrastructure limitations, and supply disruptions should be explicitly considered during preparedness planning.

### 14.3 Promote equitable and evidence-informed access to vaccines and therapeutics

National authorities, regulators, research sponsors, ethics committees, and international health organizations should avoid automatic exclusion of pregnant and breastfeeding women from potentially beneficial Ebola interventions and research. Decisions should be based on assessment of intervention-specific risks and benefits, disease severity, available evidence, and ethical considerations. Pregnancy-specific pharmacovigilance and maternal–infant outcome monitoring should accompany vaccine and therapeutic deployment whenever feasible.

These priorities represent preparedness recommendations derived from the evidence synthesized in this review and should be adapted to local epidemiological conditions, health-system capacity, available resources, and the characteristics of individual Ebola outbreaks.

## 15 Conclusion

Available evidence suggests that pregnancies complicated by symptomatic EVD pose significant maternal, fetal, and neonatal risks; however, the magnitude of these risks varies across outbreaks and is unknown due to small sample sizes, heterogeneous evidence, differences in clinical management, and limited pregnancy-specific surveillance.

National authorities, regulators, research sponsors, ethics committees, and international health organizations should avoid automatic exclusion of pregnant and breastfeeding women from potentially beneficial Ebola interventions and research. Decisions should be based on assessment of intervention-specific risks and benefits, disease severity, available evidence, and ethical considerations. Pregnancy-specific pharmacovigilance and maternal–infant outcome monitoring should accompany vaccine and therapeutic deployment whenever feasible.

Pregnancy-disaggregated surveillance, continuity of key maternity and newborn services, maternity-specific IPC readiness, and responsible access to vaccinations and medicines should all be integrated into overall outbreak planning and response. Strengthening the knowledge foundation through pregnancy-specific research and maternal–infant follow-up will also help to improve clinical and public-health decision-making during future epidemics.
